# Increasing the Time Interval between PCV Chemotherapy Cycles as a Strategy to Improve Duration of Response in Low-Grade Gliomas: Results from a Model-Based Clinical Trial Simulation

**DOI:** 10.1155/2015/297903

**Published:** 2015-12-15

**Authors:** Pauline Mazzocco, Jérôme Honnorat, François Ducray, Benjamin Ribba

**Affiliations:** ^1^Inria, Project-Team NUMED, Ecole Normale Supérieure de Lyon, 46 allée d'Italie, 69007 Lyon Cedex 07, France; ^2^Hôpital Pierre Wertheimer, 59 boulevard Pinel, 69500 Bron, France

## Abstract

*Background*. We previously developed a mathematical model capturing tumor size dynamics of adult low-grade gliomas (LGGs) before and after treatment either with PCV (Procarbazine, CCNU, and Vincristine) chemotherapy alone or with radiotherapy (RT) alone.* Objective*. The aim of the present study was to present how the model could be used as a simulation tool to suggest more effective therapeutic strategies in LGGs. Simulations were performed to identify schedule modifications that might improve PCV chemotherapy efficacy.* Methods*. Virtual populations of LGG patients were generated on the basis of previously evaluated parameter distributions. Monte Carlo simulations were performed to compare treatment efficacy across* in silico* clinical trials.* Results*. Simulations predicted that RT plus PCV would be more effective in terms of duration of response than RT alone. Additional simulations suggested that, in patients treated with PCV chemotherapy, increasing the interval between treatment cycles up to 6 months from the standard 6 weeks can increase treatment efficacy. The predicted median duration of response was 4.3 years in LGGs treated with PCV cycles given every 6 months versus 3.1 years in patients treated with the classical regimen.* Conclusion*. The present study suggests that, in LGGs, mathematical modeling could facilitate clinical research by helping to identify,* in silico*, potentially more effective therapeutic strategies.

## 1. Introduction

Diffuse low-grade gliomas (LGGs) in adults include World Health Organization (WHO) grade II astrocytomas, oligodendrogliomas, and oligoastrocytomas and account for about 25% of diffuse gliomas. LGGs are characterized radiologically by slow and continuous growth preceding anaplastic transformation [1]. Most LGGs occur in young adults between the ages of 30 and 45. Median survival ranges from 5 to 15 years [2]. Because of the relatively low incidence of LGGs, their slow growth, and the prolonged survival of patients, clinical trials in LGGs require extensive and long-lasting collaborative efforts, and clinicians must carefully select the therapeutic innovations in which to invest such efforts. Thus, clinicians stand to benefit greatly from tools that assist in identifying,* in silico*, the most relevant strategies that should be tested.

Mathematical models of tumor growth and response to treatment are being increasingly considered as relevant tools to conceive more effective therapeutic strategies [3, 4]. They provide a means of quantitatively characterizing the efficacy and toxicity of anticancer treatments [5] and can be used to optimize the efficacy of existing drugs by suggesting new scheduling regimens and relevant combination partners. For example, using an evolutionary mathematical model to investigate the emergence of acquired resistance in EGFR-mutated non-small cell lung cancer treated with erlotinib [6, 7], some authors have proposed a modification of erlotinib treatment scheduling that is expected to reduce the probability of appearance of resistant cells [8]. A phase I clinical trial was recently launched to test the efficacy of this model-based protocol (Memorial Sloan Kettering Cancer Center; Astellas Pharma US, Inc., Low Dose Daily Erlotinib in Combination With High Dose Twice Weekly Erlotinib in Patients With EGFR-Mutant Lung Cancer, in: ClinicalTrials.gov [Internet], Bethesda (MD): National Library of Medicine (USA), 2000-[2014 June 03], available from http://clinicaltrials.gov/show/NCT01967095, NLM Identifier: NCT01967095).

We previously developed a mathematical model with the capacity to describe the growth (in terms of mean tumor diameter—MTD) of adults' LGGs (most of them underwent biopsies) before and after treatment with chemotherapy or radiotherapy (RT) [9]. The aim of the present study was to assess whether this model could be used to suggest potentially more effective therapeutic strategies in LGGs. As a proof of principle, we first used the model as a simulation tool in order to perform an* in silico* clinical trial comparing the efficacy of RT plus PCV (Procarbazine, CCNU, and Vincristine) chemotherapy versus RT. In a second step, we used the model as a simulation tool to identify scheduling modifications that might improve the efficacy of the PCV chemotherapy regimen. In particular, we found that increasing the time interval between PCV cycles up to 6 months from the standard 6 weeks can potentially increase the duration of patient response.

## 2. Methods

### 2.1. Structure of the Mathematical Model of LGG Growth and Response to PCV Chemotherapy and/or Radiotherapy (RT)

The model that we previously developed [9] is based on the hypothesis that (1) LGGs are composed of two types of tissue, proliferative and quiescent tissue (with a large proportion of quiescent tissue, as confirmed by histological reports [10]) and (2) chemotherapy and RT act by inducing DNA damage in both proliferative and quiescent tissues. Proliferative cells die rapidly due to treatment-induced lesions, while quiescent cells can remain dormant for a prolonged period. The model assumes that when quiescent tumor cells receive a signal to reenter the proliferation cycle, they either repair their DNA lesions and become proliferative again or die as a result of the treatment-induced lesions. We showed that this model accurately described the evolution of tumor size before, during and after treatment in a series of 21 LGG patients treated with first-line PCV chemotherapy and in a series of 25 patients treated with first-line RT. The equations of the model and its schematic representation are provided as Supplementary Material, available online at http://dx.doi.org/10.1155/2015/297903. For the purpose of the present study, we modified the model to enable it to simulate a treatment consisting of RT followed by PCV. We distinguished the effect of RT on tumor cells from the effect of PCV and assumed that the two effects are additive. First, RT acts on proliferative and quiescent tumor cells, which respond according to the process described above. Then, PCV is administered and has an effect on the remaining proliferative and undamaged quiescent tumor cells. While PCV is taking effect, tumor cells damaged by RT continue the death (or lesion repair) process, without disruption. This means that tumor size can decrease as a result of RT effect as well as PCV effect at the same time. We can note that cells damaged by PCV have a larger capability to repair their DNA lesions than cells damaged by RT. This could lead to a faster progression of the tumor when treated by RT + PCV.

### 2.2. Simulation Method

In order to use the model as a tool for simulating patient response to RT only and to a combination of RT and PCV (RT + PCV), we generated an initial population of 1000 virtual LGG patients, characterized by a set of parameters previously identified as relevant to RT and PCV in LGG [9]. To obtain each patient's individual parameter values, we sampled population distributions of these parameters, based on analysis of data from LGG patients treated with RT only (*n* = 25) or PCV only (*n* = 21), reported in [9]. We then used Monte Carlo simulations to evaluate the effect, at a population level, of a treatment consisting either of RT or of RT plus 6 cycles of PCV; for the latter, we assumed additive effects of the two treatment modalities, as described above. The large virtual population enables representing all the tumor behaviors that could be observed within an actual population of LGG patients.

To test the effect of alternative PCV schedules, we generated a second population of 1000 virtual LGG patients, by sampling the population parameter distributions for PCV reported in [9]. For each PCV schedule that was tested, we implemented the desired frequency of treatment delivery and simulated the tumor size time course in the whole population.

The efficacy of the simulated treatments was compared using Kaplan-Meier curves, with tumor regrowth as the event. To test the significance of differences among different treatment options, we used a log-rank test applied to a subpopulation of 50 patients who were randomly sampled from the total virtual population. This subpopulation could correspond to patients enrolled in a clinical trial.

## 3. Results

### 3.1. *In Silico* Comparison of the Efficacy of RT Plus PCV versus RT in Delaying Time to Tumor Regrowth

**Figure 1:**
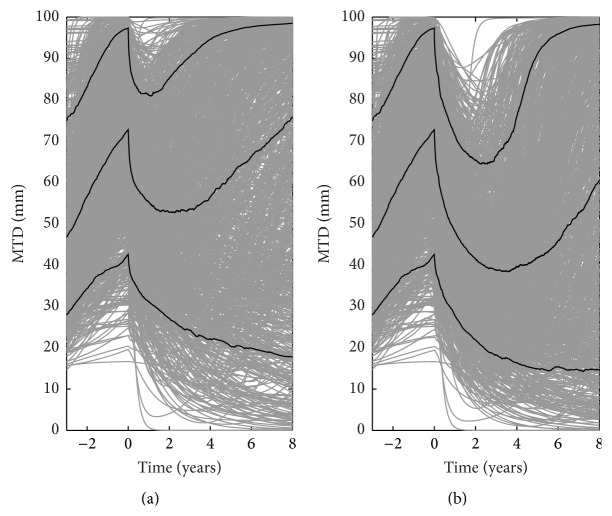
Tumor dynamics of 1000 virtual patients treated with RT or with RT plus PCV. (a) 1000 simulations of tumor size time course (MTD in mm versus time in years) for virtual LGG patients treated with RT. (b) 1000 simulations of tumor size time course for the same virtual LGG patients treated with RT followed by 6 cycles of PCV. The black lines represent the 10th, 50th, and 90th percentiles of the population dynamics.

First, we used the model as a simulation tool to compare the efficacy of RT + PCV versus RT in a population comprising 1000 virtual LGG patients (Figure 1 shows the dynamics of the population's tumor response to treatment). To this end, for each virtual patient, we computed the time to tumor regrowth as the time between treatment onset and the time when the tumor size reaches its minimum. We used the time to tumor regrowth as a metric of response duration, instead of progression-free survival (PFS), as we did not have data to support PFS modeling when we first built the model based on real patients' data.

The model predicted that time to tumor regrowth would be significantly longer in the RT + PCV arm than in the RT-only arm (*p* = 0.02, log-rank test), with a predicted median time to tumor regrowth of 4.3 years in the RT + PCV arm versus 2.9 years in the RT arm. Patients showed prolonged response to RT + PCV, as for RT, despite the larger capacity of damaged cells to repair their DNA lesions due to PCV treatment. These results are consistent with the results of a recent clinical trial (RTOG 9802), which proved the efficacy of adjuvant chemotherapy to RT. This outcome suggests that our model, which was developed on the basis of analysis of a limited series of LGG patients treated with RT only (*n* = 25) or PCV only (*n* = 21) [9], can be used to simulate tumor response in patients exposed to a combination of the two treatment types.

### 3.2. Lengthening the Time Interval between PCV Cycles Could Improve the Duration of Response

**Figure 2:**
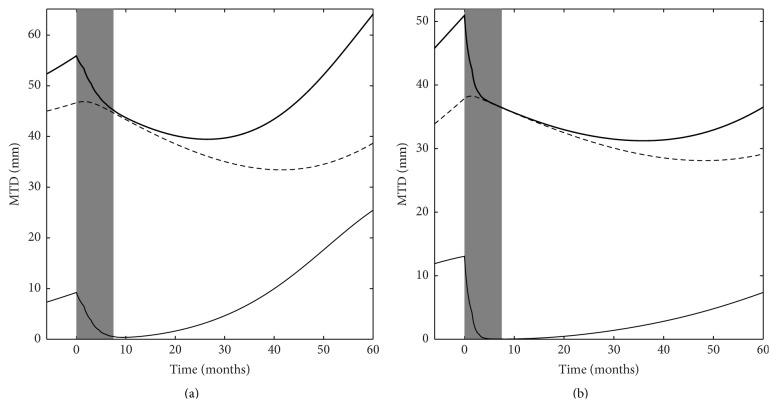
Simulation of tumor dynamics for particular patients based on model parameter estimates. (a) Simulation of tumor size time course (solid thick line) for a particular patient (patient 2 from our previous analysis [9]), characterized by the following set of parameters: *P*_0_ = 7.33 mm, *Q*_0_ = 45.01 mm, *λ*_*P*_ = 0.156 mo^−1^, *k*_*PQ*_ = 0.0334 mo^−1^, *k*_*Q*_*p*_*P*_ = 0.0039 mo^−1^, *δ*_*Q*_*p*__ = 0.0086 mo^−1^, *γ* = 1.135, and KDE = 0.29 mo^−1^ and treated with 6 cycles of PCV (grey area). The proliferative (solid thin line) and quiescent tissue (dashed line) dynamics are inferred from the MTD time course. (b) Simulation of tumor time course for a particular LGG patient (patient 3), characterized by the following set of parameters: *P*_0_ = 11.90 mm, *Q*_0_ = 33.90 mm, *λ*_*P*_ = 0.133 mo^−1^, *k*_*PQ*_ = 0.0532 mo^−1^, *k*_*Q*_*p*_*P*_ = 0.0022 mo^−1^, *δ*_*Q*_*p*__ = 0.0071 mo^−1^, *γ* = 3.046, and KDE = 0.32 mo^−1^ and treated with 6 cycles of PCV. The prolonged response is shown to be mainly due to a durable inhibition of proliferative tissue after cessation of PCV treatment.

**Figure 3:**
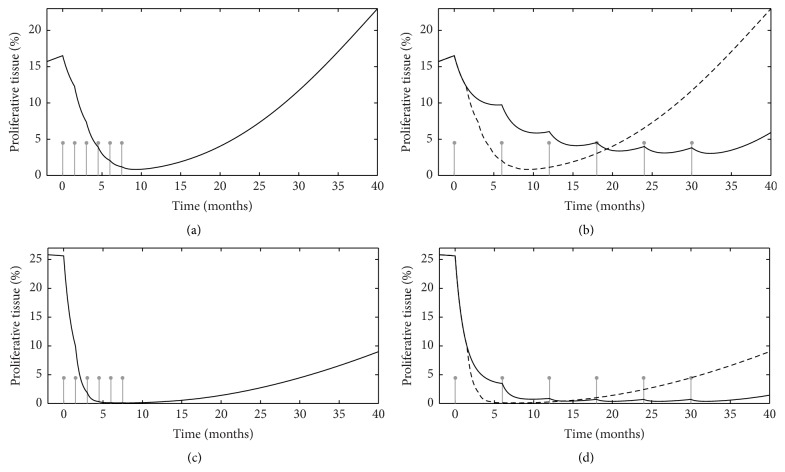
Comparison between standard and modified protocols for particular patients. (a, b) Predicted percentage of proliferative tissue—the main target of PCV chemotherapy—for patient 2, according to the time of delivery of PCV cycles indicated by the vertical lines, under the standard protocol (a) and under a modified protocol with a prolonged interval between cycles (b, solid line). (c, d) Predicted percentage of proliferative tissue for patient 3, under the standard PCV protocol (c) and under a modified protocol with a prolonged interval between cycles (d, solid line).

The second objective of our study was to use the model as a simulation tool to assess whether it might be used to suggest a potentially more effective PCV chemotherapy schedule. For this purpose we first analyzed the predicted evolution of the proliferative tissue (*P*) and the quiescent tissue (*Q*) in LGGs treated with PCV chemotherapy.  Figure 2 depicts the predicted evolution of *P*, *Q*, and *P* + *Q* (= MTD) variables in two patients (patients 2 and 3) from the set of real patients whose data were analyzed in [9]. Patient 2 was among those patients whose tumors quickly resumed growth following treatment termination (about 10 months after PCV discontinuation), whereas patient 3 was typical of patients with a prolonged decrease in tumor volume (about 30 months) after PCV discontinuation. In each patient, one can see that, according to the model, the ongoing decrease in MTD after PCV discontinuation corresponded to a decrease in *Q* (i.e., the delayed death of damaged quiescent tumor cells) that took place when *P* values were low as a result of chemotherapy. In these two patients, it appears that the duration of the MTD decrease depended on the duration of the inhibition of the growth of the proliferative cells (short in patient 2, prolonged in patient 3). Therefore, the model logically suggests that, to increase the duration of response to PCV chemotherapy, the duration of *P* inhibition must be increased. The most straightforward method to prolong *P* inhibition would be to prolong the duration of chemotherapy, but this is not possible with the PCV regimen given its cumulative toxicity. However, observing the evolution of *P* in patient 3 led us to hypothesize that another way to prolong *P* inhibition may be to lengthen the time interval between PCV cycles so that each PCV cycle would be given when it can have maximal efficacy.  Figure 3 shows the predicted change in MTD evolution in patients 2 and 3 when the time between two PCV cycles is lengthened to 6 months from the standard 6 weeks. In patient 3, the model indicates that, under the standard PCV regimen, *P* already becomes very small after the first three PCV cycles, such that the last three PCV cycles are delivered while the percentage of proliferative cells is already at a minimal level (Figure 3(c)). Therefore, it is possible that the potential efficacy of the last three PCV cycles may be “wasted” somewhat.

**Figure 4:**
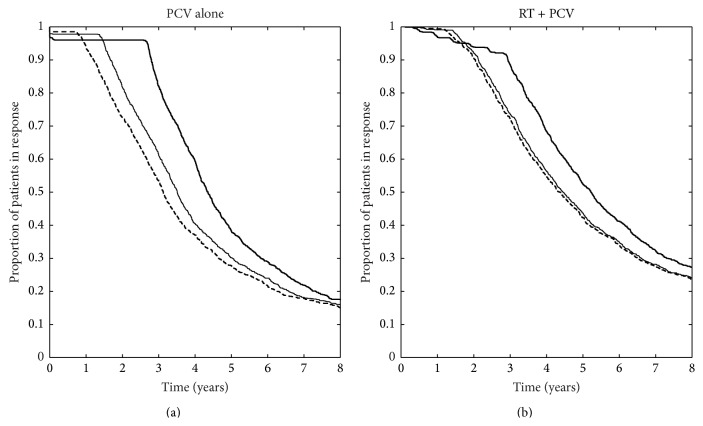
Comparison between different therapeutic protocols for the virtual populations. (a) Kaplan-Meier curve of the time to tumor regrowth in virtual patients treated with the standard (thick dashed line) and the prolonged (thick solid line) PCV protocol. Thin line represents the Kaplan-Meier curve of the time to tumor regrowth for patients treated with an intermediate protocol: 6 PCV cycles every 3 months. (b) Kaplan-Meier curve of the time to tumor regrowth in virtual patients treated with RT plus standard PCV (dashed line) or RT plus modified PCV with a 6-month interval between cycles (solid line). Thin line represents the Kaplan-Meier curve of the time to tumor regrowth for patients treated with RT plus 6 cycles of PCV administered every 3 months.

To test the hypothesis that increasing the interval between PCV cycles would increase the duration of LGG response, we used the model parameters obtained in our series of patients treated with PCV only [9] to create a virtual population of 1000 LGG patients and simulated different PCV schedules in this population. We assumed that each new PCV cycle has the same effect as the previous one, as there is no evidence—to our knowledge—of emergence of resistance due to PCV chemotherapy. As shown in Figure 4(a), the model predicted that, in patients treated with first-line PCV chemotherapy, increasing the time interval between cycles up to 6 months from the standard 6 weeks was associated with a significant increase in the duration of the response (*p* = 0.0028, log-rank test). The median duration of response was 4.3 years in LGGs treated with PCV cycles given every 6 months versus 3.1 years in patients treated with the classical regimen. Lengthening the interval beyond 6 months was associated with tumor regrowth between cycles. Finally, we tested in our virtual population whether lengthening the time interval between PCV cycles might also be beneficial in patients who receive adjuvant PCV after RT. As shown in Figure 4(b), in this situation, the duration of response was significantly longer in patients treated with the PCV-6 months' schedule than in patients treated with the classical PCV schedule (*p* = 0.035, log-rank test). Median time to tumor regrowth was also longer (5.2 years versus 4.3 years, resp.). The outcomes with an intermediate treatment protocol (PCV cycles administered every 3 months for PCV alone and RT plus PCV) are also displayed in Figure 4. Expected percentages of patients still in response 2 years, 3 years, and 4 years after treatment onset, for each treatment protocol, are presented in Table 1.

## 4. Discussion

LGGs are relatively rare, slow-growing tumors. As a result, clinicians have limited opportunities to carry out clinical trials in LGG and must design such trials carefully. In the present study, we have shown how mathematical modeling can be used to suggest more effective therapeutic strategies and facilitate clinical research in LGGs.

First, we performed an* in silico* clinical trial comparing RT versus RT + PCV and showed that though the model was developed on the basis of data from a limited series of patients treated with RT alone or PCV alone, model simulations suggested that RT + PCV would be more effective than RT alone in terms of duration of response. This prediction is consistent with the results of the RTOG 9802 trial [11], although we note that the latter trial measured progression-free survival, which, of course, is not identical to our measurement of duration of response (based on tumor volume assessment). Despite the difference between the results we obtained with the simulations and the results from the clinical trial, our results suggest that once the effects of two treatments have been modeled separately, simulations can be performed to evaluate the efficacy of combining these two treatments. For example, modeling the effect of temozolomide and RT in the ongoing EORTC trial comparing these two treatments could provide an indication of the efficacy of treating LGG patients with RT plus adjuvant temozolomide [12].

Our model also enabled us to simulate the potential impact of modifying a current standard LGG treatment schedule. Though LGGs are slow-growing tumors in which the doubling time of proliferative tissue is very long, they are treated with chemotherapy schedules developed to treat high-grade gliomas, which are aggressive, fast-growing tumors. Our model simulations suggested that the classic 6-week interval between PCV chemotherapy cycles might not be optimal in LGGs and that longer time intervals between PCV cycles (up to 6 months) would increase the duration of response due to increased impact of the treatment on the proliferative tissue. Simulations further indicated that increasing the intercycle interval beyond 6 months would lead to tumor growth between cycles. Increasing the time intervals between cycles might also have the advantage of reducing PCV hematotoxicity.

Optimizing chemotherapy efficacy in LGGs is an important issue. In progressive, symptomatic, or high-risk LGGs requiring treatment other than surgery, the results of a recent phase III study comparing radiotherapy (RT) alone to RT plus adjuvant PCV chemotherapy have shown that RT plus PCV results in significantly higher median overall survival compared with RT alone [13].

Yet, owing to their prolonged overall survival, LGG patients treated with RT plus PCV are at risk of delayed radiation-induced cognitive dysfunction, and first-line chemotherapy might be an interesting strategy to safely defer RT in order to reduce the risk of neurocognitive deterioration [14]. Several studies have shown that LGGs can respond to first-line chemotherapy; however many questions remain unanswered regarding the optimal scheduling and duration of such treatment [15]. Our model simulations suggest that increasing the time interval between cycles might be a simple way of increasing PCV chemotherapy efficacy. These predictions, however, remain to be validated in a prospective study.

In the present study, we have proposed a protocol consisting of administering six PCV cycles separated by 6-month intervals. The total duration of treatment under this protocol is four times longer than that under the standard protocol. In our simulations, this schedule led to a significant increase in tumor response duration in most virtual patients. It therefore seems that it would be worthwhile to carry out a clinical trial that tests the effects of prolonging the time interval between PCV cycles, using simulation methods to determine an optimal PCV schedule. The time interval between cycles should be chosen carefully to prevent tumor progression during treatment. One option might be to test a schedule in which the first two PCV cycles are given with a 6-week interval to achieve major tumor reduction, and the four remaining cycles are given every 6 months to prolong the effect of the therapy. Our simulation results also suggest that prolonging the interval between PCV cycles when given in combination with RT should also result in improving regimen efficacy although to a less extent.

Though the results of the present study are promising, it is necessary to exercise great caution when attempting to translate the results of a mathematical model into therapeutic recommendations. One limitation of our model is that it only describes the evolution of tumor diameter, which is* per se* of limited clinical relevance. To improve clinical accuracy and reliability of our results, we could think of using volumetric measures instead of observations based on tumor diameter measures. Indeed, because low-grade gliomas slowly diffuse in the brain, unusual shaped lesions can be observed and may be better captured by tumor volumetric measures than by tumor diameter measures. In addition, the molecular characteristics of LGGs, as the 1p/19q codeletion or IDH mutation, are not taken into account. These limitations are due to the limited number of patients whose data were analyzed to develop the model; we are confident that analyzing a larger series of patients with available molecular data would enable us to develop a model integrating molecular characteristics of LGG and linking the evolution of the MTD to overall survival. Despite these limitations, we propose that the quantitatively based framework we have developed can provide relevant, data-driven insights regarding means of improving chemotherapy efficacy, especially in settings in which schedules are determined primarily on the basis of empirical considerations.

## Supplementary Material

Supplementary material provides a description of the model used in this study, with its schematic representation and mathematical equations. This model was analyzed through a population approach, using mixed-effects modeling. Model parameters were estimated using the full time-course of tumor size in 21 patients treated with PCV. The estimates are reported in the Supplementary material.

## Figures and Tables

**Table 1:** Expected percentages of patients still in response after treatment onset for the different protocols tested.

	PCV alone			RT + PCV		
Years after treatment onset	Standard protocol	3-month protocol	6-month protocol	Standard protocol	RT + 3-month PCV protocol	RT + 6-month PCV protocol
2 years	72.6%	81.8%	96%	90.5%	92.1%	93.9%
3 years	53.6%	61.5%	82.2%	72.1%	73.7%	88.6%
4 years	37.1%	40.5%	59.5%	54.7%	56.3%	68.4%
