# Supplementary material for: Increasing the Time Interval between PCV Chemotherapy Cycles as a Strategy to Improve Duration of Response in Low-Grade Gliomas: Results from a Model-Based Clinical Trial Simulation
Source: Comput Math Methods Med. 2015 Dec 15;2015:297903. doi: 10.1155/2015/297903 (PMC4693002; doi:10.1155/2015/297903)
Supplement: Supplementary file 1 — Supplementary material provides a description of the model used in this study, with its schematic representation and mathematical equations. This model was analyzed through a population approach, using mixed-effects modeling. Model parameters were estimated using the full time-course of tumor size in 21 patients treated with PCV. The estimates are reported in the Supplementary material. [file 297903.f1.pdf]

Supplementary Material

The figure displays a schematic representation of the model developed for the analysis of LGG tumor size measurements.

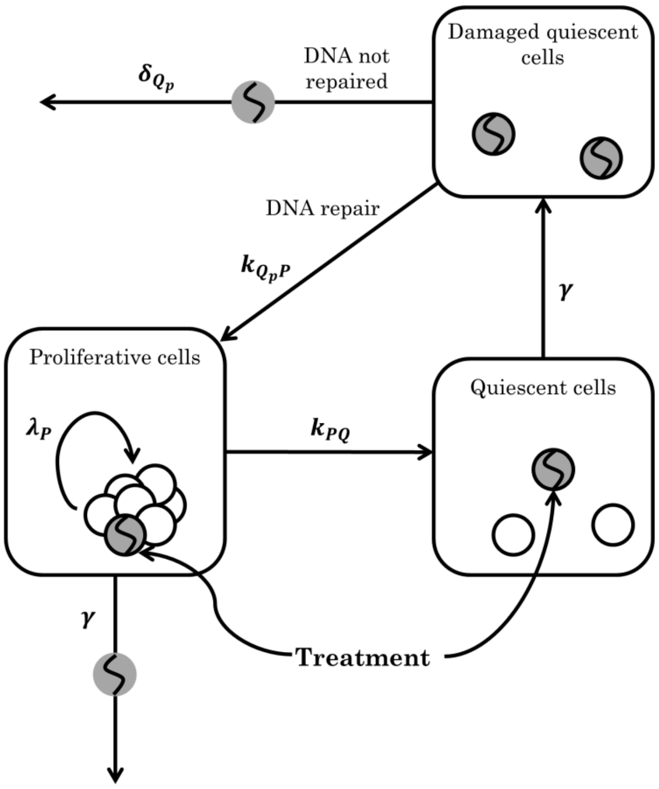

The full mathematical equations of the model are:

$$\frac{dC}{dt} = -KDE \times C$$

$$\frac{dP}{dt} = \lambda_P \times P \times \left(1 - \frac{P^*}{100}\right) + k_{Q_pP} \times Q_p - k_{PQ} \times P - \gamma \times C \times KDE \times P$$

$$\frac{dQ}{dt} = k_{PQ} \times P - \gamma \times C \times KDE \times Q$$

$$\frac{dQ_p}{dt} = \gamma \times C \times KDE \times Q - k_{Q_pP} \times Q_p - \delta_{Q_p} \times Q_p$$

$$P^* = P + Q + Q_p$$

P denotes the density of proliferative tissue, and Q the density of quiescent tissue that can become DNA-damaged (denotes Qp) due to the effect of the chemotherapy. The sum $P + Q + Q_p$,

calculating by solving the system of ordinary differential equations presented above was directly compared to the measured MTD in patients to estimate the typical value of model parameters.

We modeled the pharmacokinetics of the PCV chemotherapy using a kinetic–pharmacodynamic approach, in which drug concentration is assumed to decay according to an exponential function [1]. In the model, we did not consider the 3 drugs separately. Rather, we assumed the treatment to be represented as a whole by a unique variable (C), which represents the concentration of a virtual drug encompassing the 3 chemotherapeutic components of the PCV regimen. We modeled the exact number of treatment cycles administered by setting the value of C to 1 (arbitrary unit) at the initiation of each cycle T_{treat}: $C(t = T_{treat}) = 1$. As a result, not the exact dose of PCV chemotherapy was integrated but the exact time (scheduling) of PCV administration was.

The model is composed by 8 parameters to be estimated including 2 initial conditions (initial estimates of the size of both proliferative and quiescent tissue). The model parameters were described by random variables (mixed-effect or population approach [2]) to characterize the variability observed in MTD growth and response to treatment in the population of patients. Variability was assumed on all model parameters except the KPD parameter related to the kinetic of drug concentration in plasma. Individual parameters were log-normally distributed, i.e. for an individual parameter ψ_i, $\psi_i = \psi \times \exp(\eta_i)$ where ψ is the typical (population) value of the parameter and η_i representing the contribution of the individual i. η_i are normally-distributed with mean 0. Both population and individual parameters were estimated with the SAEM (Stochastic Approximation of the Expectation Maximization) algorithm implemented in Monolix 4.2 (Lixoft) using the full MTD time-course in the 21 patients. Parameters were also estimated for radiotherapy and temozolomide (see [3] for further details). We used the following parameter estimates:

		PCV		Radiotherapy	
Parameters	Units	Mean value (rse)	CV (%) (rse)	Mean value (rse)	CV (%) (rse)
P_0	mm	7.13 (25%)	94 (23%)	3.89 (28%)	67 (68%)
Q_0	mm	41.2 (7%)	54 (10%)	40.3 (6%)	49 (12%)
λ_P	mo-1	0.121 (16%)	72 (9%)	0.138 (16%)	62 (18%)
k_{PQ}	mo-1	0.0295 (21%)	76 (12%)	0.0249 (41%)	89 (28%)
k_{Q_pP}	mo-1	0.0031 (35%)	97 (31%)	-	-
δ_{Q_p}	mo-1	0.00867 (21%)	75 (12%)	0.0125 (29%)	97 (18%)
γ	-	0.729 (37%)	115 (9%)	1.71 (24%)	83 (20%)
KDE	mo-1	0.24 (33%)	70 (FIXED)	0.317 (60%)	70 (FIXED)

Supplementary references

1. Jacqmin P, Snoeck E, van Schaick E a, Gieschke R, Pillai P, Steimer J-L, et al. Modelling response time profiles in the absence of drug concentrations: definition and performance evaluation of the K-PD model. J Pharmacokinet Pharmacodyn. 2007;34: 57–85. doi:10.1007/s10928-006-9035-z

2. Lindstrom M., Bates D. Nonlinear mixed effects models for repeated measures data. Biometrics. 1990;46: 673–87.

3. Ribba B, Kaloshi G, Peyre M, Ricard D, Calvez V, Tod M, et al. A Tumor Growth Inhibition Model For Low-Grade Glioma Treated With Chemotherapy or Radiotherapy. Clin Cancer Res. 2012;18: 5071–80. doi:10.1158/1078-0432.CCR-12-0084
